# Macrophage and Innate Lymphoid Cell Interplay in the Genesis of Fibrosis

**DOI:** 10.3389/fimmu.2015.00597

**Published:** 2015-11-23

**Authors:** Emily Hams, Rachel Bermingham, Padraic G. Fallon

**Affiliations:** ^1^Trinity Biomedical Sciences Institute, School of Medicine, Trinity College Dublin, Dublin, Ireland

**Keywords:** fibrosis, macrophages, innate lymphoid cells, Th2 cells, epithelial-derived cytokines

## Abstract

Fibrosis is a characteristic pathological feature of an array of chronic diseases, where development of fibrosis in tissue can lead to marked alterations in the architecture of the affected organs. As a result of this process of sustained attrition to organs, many diseases that involve fibrosis are often progressive conditions and have a poor long-term prognosis. Inflammation is often a prelude to fibrosis, with innate and adaptive immunity involved in both the initiation and regulation of the fibrotic process. In this review, we will focus on the emerging roles of the newly described innate lymphoid cells (ILCs) in the generation of fibrotic disease with an examination of the potential interplay between ILC and macrophages and the adaptive immune system.

## Introduction

Fibrosis is a characteristic pathological feature of an array of chronic diseases. The development of fibrosis in distinct tissues and organs is associated with numerous conditions, for example, idiopathic pulmonary fibrosis (IPF), cystic fibrosis (CF), systemic sclerosis, non-alcoholic steatohepatitis (NASH), primary biliary cirrhosis, cancer, and atherosclerosis. In these diseases, the chronic development of fibrosis in tissue can lead to marked alterations in the architecture of the affected organs and subsequently cause defective organ function. As a result of this process of sustained attrition to organs, many diseases that involve fibrosis are often progressive conditions and have a poor long-term prognosis. Indeed, due to the limited understanding of the mechanisms underlying the generation of fibrosis and the heterogeneity of fibrotic disease, there is currently a paucity of effective treatment strategies, contributing to the poor prognosis. The processes that underlie fibrosis are a tightly controlled natural mechanism of repair; however, dysregulation in the wound healing mechanism can result in aberrant fibrosis. Inflammation is often a prelude to fibrosis, with innate and adaptive immunity involved in both the initiation and regulation of the fibrotic process. In different organs, the insult to distinct cells, for example, bronchial epithelial cells in the respiratory tract, can lead to cell damage and release of various mediators, such as damage-associated molecular patterns (DAMPs), as well as proinflammatory and profibrotic factors. The mediators released can, depending on prevailing stimuli and local cellular environment, initiate a cascade within the cellular milieu in a tissue that leads to the accumulation of extracellular matrix components (ECM), rich in fibrillar collagens, fibronectin, and hyaluronic acid culminating in the deposition of fibrous connective tissue (1, 2). In this review, we will focus on the development of pulmonary fibrosis and the emerging roles of the newly described innate lymphoid cells (ILCs) in the generation of fibrotic disease with an examination of the potential interplay between ILC and macrophages.

## Inflammation and Fibrosis

While chronic injury is a prominent factor in many fibrotic diseases, acute inflammatory reactions may also play an important role in the initiation of fibrosis. Using experimental models involving acute lung injury, such as bleomycin-induced pulmonary fibrosis, where cellular apoptosis and necrosis are the underlying causative mechanisms, acute inflammatory responses initiated via activation of DAMP signaling cascades, results in a profibrotic response. While most chronic fibrotic diseases have an underlying inflammatory cause in many cases, for example, IPF, the causative mechanisms are not fully understood. Indeed, IPF is not responsive to anti-inflammatory steroid treatment, conversely treatment appears to exacerbate disease (3). However, in certain fibrotic disorders where the inflammatory cause has been identified, the use of anti-inflammatory therapies, such as ibuprofen to reduce the symptoms of CF (4, 5), demonstrate the potential roles of inflammation in chronic fibrotic diseases.

A loss of membrane integrity of cells, through injury, apoptosis, or necrosis, results in uncontrolled release of cellular contents, some of which can act as DAMPs, initiating an inflammatory response to clear cellular debris and initiate wound healing. In addition, DAMPs can be further synthesized and released in response to local cellular damage. The receptors for DAMPs, the pattern recognition receptors, including the Toll-like receptor (TLR) family, can in addition to recognizing pathogen-associated molecular patterns (PAMPs) identify fragments of ECM, such as hyaluronic acid and fibrinogen cleavage products (6). Indeed, effective danger signaling is implicated in the generation of fibrosis, with TLR2-, TLR3-, TLR4-, and TLR9-deficient animals demonstrating exacerbated collagen deposition in experimental disease models (7). The excessive synthesis and release of DAMPs underlies “sterile inflammation,” with innate immune cells promoting inflammation in the absence of an active infection (8). Apoptotic and necrotic epithelial cells are a primary source of DAMPs, in particular ATP, IL-33, and uric acid that can initiate fibrosis (7). Release of uric acid, which crystallizes locally, can activate the NALP3 inflammasome in macrophages resulting in the release of IL-1β (9). Inflammasome activation leads to an increase in a number of other proinflammatory and profibrotic cytokines and chemokines, such as CXCL1, platelet-derived growth factor (PDGF), and transforming growth factor β1 (TGF-β1), linking innate immune activation and generation of fibrosis (10). Due to the requirement for inflammasome activation in the processing of IL-1β and IL-18 and the upregulation of other profibrotic mediators, inflammasome activation may play a critical role in wound healing; however, further investigation is required to address the potential for therapeutics targeting the inflammasome as beneficial in fibrotic disease.

## The Epithelial Barrier in Wound Healing and Fibrosis

The epithelium serves as the initial defense against insult, providing both a physical and mechanical barrier, and is therefore a crucial interface to orchestrate both the innate and adaptive immune responses. Proinflammatory mediators released by damaged and dying epithelial cells, as well as recruited leukocytes, activate mesenchymal precursor cells in tissues and induce their trans-differentiation to ECM-producing myofibroblasts (1). The fibrosis cascade progresses following the insult to cells and subsequent release of mediators, such as IL-13, connective tissue growth factor (CTGF), and TGF-β, that operates downstream of initial cellular injury (6, 11). The mature epithelium in the lung is non-proliferative; however, in response to injury or inflammation, it is vital that the damage to the epithelium is repaired to ensure it remains an effective physical barrier. The signaling pathways activated in the process of repairing epithelial damage are similar to those initiated during development, with the dysregulation of these developmental pathways underlying the generation of fibrosis (12).

Transforming growth factor-β is the major profibrotic cytokine; it has central roles in promoting the activation and proliferation of fibroblasts, upregulates α-smooth muscle actin (α-SMA) and collagen I synthesis by myofibroblasts and promotes epithelial-to-mesenchymal transition (EMT) (6). The Wnt signaling pathway has also been implicated in EMT, with overexpression of the WNT-1 inducible signaling protein regulating the expression of profibrotic markers, such as MMP7 and plasminogen-activator inhibitor 1 (PAI-1), thus promoting EMT locally (6). CTGF is a matricellular protein, which can mediate the activities of a number of other profibrotic and angiogenic factors, such as TGF-β, bone morphogenic protein (BMP) 4, and vascular endothelial growth factor (VEGF) (13, 14). CTGF has been implicated in fibrosis in the liver, lung, skin, and kidney and acts synergistically with TGF-β to promote chronic fibrosis inducing ECM expression and collagen production by fibroblasts (6). Indeed, trials of antibodies targeting CTGF are currently ongoing in patients with IPF and liver fibrosis (15). Repetitive cycles of epithelial damage and repair are required for the generation of fibrosis (16, 17), with factors that damage the epithelium and initiate DAMPs and alarmin responses being actively pursued as potential therapeutic targets.

## Epithelial-Derived Cytokine Mediators of Fibrosis

In addition to the “classic” profibrotic mediators, such as TGF-β and CTGF, recent research has focused on epithelial-derived type 2 cytokines as potential therapeutic targets for fibrosis. In response to epithelial cell injury, the alarmin cytokines IL-25, IL-33, and TSLP are released and are responsible for the initiation of a cascade of inflammatory responses. These cytokines have important roles in type 2 immunity, in particular in helminth infection and allergy (18). In the context of fibrosis, all three epithelial cell-derived cytokines have individually been shown to be involved in different aspects of fibrosis and are dysregulated in patients with fibrotic diseases (Table 1).

**Table 1:** **Expression of selected epithelial-derived cytokines in human fibrotic diseases**.

Cytokine	Disease	Observation	Reference
IL-25	IPF	Increased IL-25 detected in BAL fluid of IPF patients’ levels positively correlate with fibrotic marker periostin	Hams et al. (21)
	Asthma	Rhinovirus-induced IL-25 exacerbates asthma attacks	Beale et al. (25)
	Systemic sclerosis	Increased IL-25^+^ cells in the skin of SSc patients	Lonati et al. (23)
IL-33	IPF	IL-33 is elevated in the lungs and BAL of IPF patients	Luzina et al. (34)
	Asthma	Increased IL-33 in the serum and sputum of patients with allergic asthma	Hamzaoui et al. (31)
			Guo et al. (32)
	Hepatitis	IL-33 is increased in the endothelial cells from livers of patients with hepatitis B, hepatitis C, and cirrhosis	Marvie et al. (33)
	Systemic sclerosis	Serum IL-33 is increased in SSC patients	Yanaba et al. (30)
		Serum IL-33 positively correlates with skin lesions	
TSLP	Asthma	Bronchial and BAL expression of TSLP increased in asthmatics	Ying et al. (41)
		TSLP promotes airway remodeling in lung fibroblasts	Wu et al. (42)
	Systemic sclerosis	TSLP is upregulated in the skin of patients with SSc	Christmann et al. (43)

## IL-25

IL-25, also known as IL-17E, is a member of the IL-17 family of cytokines and is secreted by many immune cells including activated Th2 cells, eosinophils, mast cells and macrophages, in addition to epithelial cells. IL-25 binds to IL-17RB, which forms a receptor complex with IL-17RA, activating the NF-κB pathway and initiating Th2-mediated inflammation. IL-25 has been implicated in both experimental models of fibrosis and has been detected in samples from patients with chronic lung conditions and in the skin of patients with systemic sclerosis (19–23). Mice deficient in IL-25, or its functional receptor IL-17RB, show impaired collagen deposition in response to bleomycin-induced lung injury or *S. mansoni* egg-induced granulomatous pulmonary inflammation (21). Furthermore, intranasal administration of IL-25 induces collagen deposition and TGF-β and CTGF expressions in the lungs (21, 22). IL-25 is also upregulated in asthma and has been shown to play a role in airway remodeling and angiogenesis both *in vitro* and in *in vivo* models (24, 25). Treatment with an anti-IL-17RB antibody, thereby blocking IL-25-mediated signaling, improves airway hyper-responsiveness in a mouse model of allergic lung inflammation (26, 27). The therapeutic benefits of inhibiting IL-25 in conditions, such as allergic lung inflammation, where airway remodeling is a key event, suggest that IL-25 is an important mediator of tissue regeneration and consequently fibrosis in conditions, such as asthma.

IL-25-dependent fibrosis elicited in the lungs has been attributed to a downstream pathway involving IL-25-mediated expansion of ILC2 within the lungs with subsequent induction of fibrosis via an IL-13-dependent mechanism (21). Further mechanistic studies have demonstrated that in addition to activating ILC2, IL-25 can also directly drive polarization of bone marrow-derived macrophages *in vitro* toward a type 2 phenotype, with increasing surface expression of M2 marker CD206, in synergy with coadministered IL-4 (28). In addition, IL-25 can directly bind to human pulmonary fibroblasts through its receptor IL-17RB and can promote proliferation and differentiation to a myofibroblastic phenotype (22). These data suggest that IL-25 is an important mediator of fibrosis with roles in human fibrotic disease and, as such, is an exciting therapeutic target.

## IL-33

IL-33 is the functional ligand for the IL-1 receptor family member ST2 in a complex with IL-1R accessory protein (IL1RAP) (29). IL-33 is not normally secreted, instead it is found localized to heterochromatin in the nucleus; however, it is released upon cell damage as an alarmin. IL-33 and ST2 have been causally linked with fibrotic conditions, including Crohn’s disease, pulmonary, and liver fibrosis (Table 1) (30–32). In mouse studies, *Il33*^−/−^ and *Il1rl1*^−/−^ mice demonstrate decreased collagen deposition in models of lung, liver, and intestinal fibrosis (33–37). Interestingly, only the full length but not the proteolytically cleaved mature IL-33 is implicated in the pathogenesis of the bleomycin-induced model of pulmonary fibrosis (34, 38). Mechanistically, IL-33 initiates a local inflammatory response through the recruitment and activation of type 2-associated effector cells including eosinophils, basophils, mast cells, and ILC2, resulting in the release of Th2 cytokines and activation of macrophages, thereby potentially contribution to the downstream development of fibrosis. Indeed, in the liver and lung, the profibrotic effects of IL-33 are closely linked with increased IL-13 production from ILC2 (35, 39, 40).

## TSLP

TSLP is secreted predominantly by keratinocytes but is also found in the small airway and intestinal epithelium, and signals via a heterodimeric receptor comprising one chain of IL-7Rα and one chain of TSLPR. TSLP has also been implicated in several models of fibrosis [Table 1 (41–43)], with diminished pulmonary and skin fibrosis in mice deficient in the receptor for TSLP (44, 45).

While it is evident that these epithelial alarmin cytokines individually contribute to the generation of fibrosis, there is overlap and functional redundancy in IL-25, IL-33, and TSLP potentially due to the ability of all three cytokines to activate ILC2, as reported by Locksley and colleagues, with respect to chitin-elicited pulmonary inflammation (46). However, this apparent redundancy may be due to different ligand and receptor expression at different anatomical sites and a hierarchy of action at each tissue, although this speculation would need experimental clarification.

## Innate Lymphoid Cells

Innate lymphoid cells are a recently described group of innate cells of a lymphoid lineage that do not express antigen-specific receptors. These cells have important roles in the innate response, regulation of homeostasis and inflammation, and interplay with adaptive immunity. While relatively rare in the systemic circulation in comparison to other hematopoietic cells, ILCs are enriched at epithelial barrier surfaces and act as regulators of chronic inflammation and tissue remodeling, acting to bridge innate and adaptive immunities.

**Figure 1:**
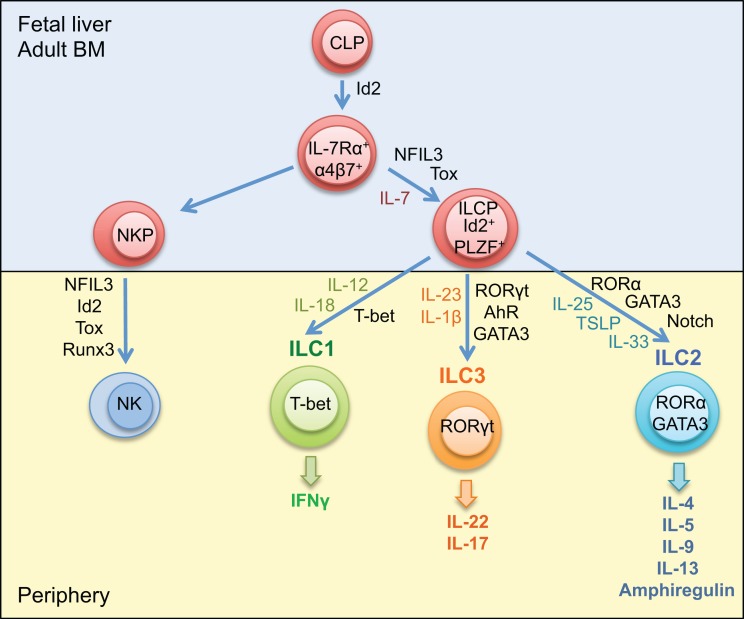
**Development of innate lymphoid cells**. Innate lymphoid cells differentiate from common lymphoid progenitors in the fetal liver or adult bone marrow. The ILC precursor develops from CLP under the influence of the transcription factors Id2, PLZF, NFIL2, and Tox. ILC1, ILC2, and ILC3 differentiate from ILCP dependent on T-bet, RORα, and GATA3, and RORγt, respectively. Maturation and activation of ILC1 requires IL-12 and IL-18; ILC2 requires IL-25, IL-33, and TSLP and the influence of the Notch signaling pathway; ILC3 requires IL-23 and IL-1β and the additional influence of the transcription factor AhR. NK cells develop from NK precursors in the bone marrow under the influence of the transcription factors Id2, NFIL3, Tox, and Runx3. CLP, common lymphoid progenitor; ILCP, innate lymphoid cell progenitor; NKP, natural killer cell progenitor; Id2, inhibitor of DNA binding 2; PLZF, promyelocytic leukemia zinc finger protein; RORα, RAR-related orphan receptor α; AhR, aryl hydrocarbon receptor.

Mature ILCs can be identified by a lack of markers associated with cells of a lymphoid lineage; however, they share expression of Thy1, the common gamma chain (γc), and IL-7Rα (47). ILC develops from common lymphoid progenitors (CLPs) in the fetal liver and adult bone marrow, relying upon the transcription factors’ inhibitor of DNA binding 2 (Id2), nuclear factor interleukin-3 regulated (NFIL3), promyelocytic leukemia zinc finger protein (PLZF), and thymocyte selection-associated mobility group box (Tox) (47–52). Expression of Id2 is essential for the development of ILCs; however, PLZF is only transiently expressed in the early ILC precursor populations, with levels barely detectable in mature ILCs, suggesting that its importance in the early development of ILCs (49). Expression of NFIL3 and Tox is detected earlier than Id2 in the development cascade of ILC; however, these transcription factors do not appear to be as critical as Id2 for ILC development, with only minimal effects observed in the ILC repertoire in mice deficient in either NFIL3 or Tox (48, 51). These precursor cells differentiate to NK precursors or common helper innate lymphoid precursors, which, under the influence of additional transcription factors and cytokines give rise to mature ILC subsets (Figure 1) (53, 54).

Innate lymphoid cells can be divided into distinct subsets based on the cytokines they produce and the transcription factors necessary for their development and function: group 1, which produces interferon (IFN) γ and includes NK cells; group 2, which produces Th2-associated cytokines; and group 3, which produces IL-17 and IL-22 (Figure 1). Expression of the transcription factors T-bet, GATA3, and RORγt is required for the development of ILC1, ILC2, and ILC3 respectively (Figure 1). While GATA3 is required for the maturation of all ILC subsets, it is expressed at much higher levels in ILC2. The transcription factors RORα and Bcl11b are also required for effective function of ILC2, deficiency in either RORα or Bcl11b diminishes the generation of mature ILC2 (55–58). Expression of the aryl hydrocarbon receptor (Ahr) appears critical for ILC3 function, with reduced IL-22 production and decreased presence of ILC3 in the intestines of *Ahr*-deficient mice (59). There is some plasticity between ILC subsets, ILC3 can downregulate RORγt expression, allowing T-bet to become the prominent transcription factor, and ILC3 cells can take on a more ILC1 phenotype associated with increased IFNγ expression (47). A recent study has also demonstrated that CD14^+^ DCs in the intestine of Crohn’s disease patients promote polarization of ILC3 to CD127^+^ ILC1 (60). An IL-25-elicited ILC2 population also has been detected, which has been shown to transition to produce IL-17 (61). Furthermore, in the absence of the T cell-associated transcription factor Bcl11b in ILC2, there is an increase in the expression of RORγt, and the cells take on an ILC3-like phenotype (57, 58, 62). While each ILC subset has unique roles in host defense and development, the plasticity between groups suggests that ILC subtypes may change depending on the tissue environment.

Innate lymphoid cells play an important role in orchestrating acute inflammation in response to infection and also chronic inflammation and wound healing. While ILC2 is commonly associated with chronic tissue inflammation and fibrosis, ILC1 has not yet been formally implicated in the pathogenesis of fibrosis, while ILC3 is also associated with the development of fibrosis and is elevated in the bronchoalveloar lavage (BAL) fluid of asthma patients (63, 64). ILC3 is an important source of IL-17, which may mechanistically underlie a role for ILC3 in fibrosis. IL-17A has been implicated in the generation of fibrosis, with elevated levels detected in patients with IPF and CF (65, 66). Furthermore, IL-17A has a critical role in the generation of bleomycin-induced pulmonary fibrosis, which is dependent on TGF-β, suggesting codependent roles for IL-17A and TGF-β in the pathogenesis of fibrosis (65). Therefore, as a source of IL-17 in mucosal tissues, ILC3 may represent an important cell subset in the progression of IL-17-mediated fibrosis. The relative roles of ILC subsets may have further implications in the pathogenesis of lung inflammation. Indeed, a recent study has identified both Th2-high and Th17-high clusters of asthma patients, which are inversely correlated (67). Experimental models have shown that therapeutically targeting one cluster promotes the other subtype and that combination therapy may prove more effective (67). This study clearly demonstrates the interplay between Th2-cytokine-producing cells and IL-17-producing cells and the potential implications for inflammatory and fibrotic diseases.

## Type 2 Innate Lymphoid Cells, Chronic Tissue Inflammation, and Fibrosis

ILC2 is characterized by their ability to produce the Th2 cytokines IL-4, IL-5, IL-9, IL-13, and amphiregulin (Figure 1) (68–71). They rely upon the transcription factors GATA3 and RORα for their development and the cytokines IL-25 and IL-33 for their maturation and recruitment (55, 69, 72). Recently, it has been reported that ILC2 can be further classified into two distinct subtypes: the IL-33-elicited Lin^-^T1/ST2^+^ “natural ILC2” (nILC2) and the IL-25-elicted Lin^-^KLRG1^hi^ “inflammatory ILC2” (iILC2) (61). While ILC2 has been implicated in the pathogenesis of fibrosis, the relative functions of nILC2 and iILC2 with regards to inflammation, tissue repair, and fibrosis has yet to be fully elucidated.

ILC2 is implicated in the effective resolution of helminth infection, and in the development of allergic inflammation (73). Furthermore, ILC2 has been shown to play an important role in wound healing, tissue repair, and consequently chronic tissue inflammation and fibrosis (74). Studies have demonstrated that while the pathogenesis of ILC2 in fibrosis is associated with IL-13 release (21, 56), ILC2-mediated wound healing and tissue regeneration in the lung are promoted by release of amphiregulin by ILC2 (70, 71). ILC2 is associated with tissue fibrosis in experimental models, and dysregulated ILC2 responses have been detected in samples from patients with chronic inflammatory diseases, including IPF, atopic dermatitis, chronic rhinosinusitis, and asthma (21, 75–78). Furthermore, depletion of ILC2 in experimental models of fibrosis attenuates collagen deposition; conversely, transfer of ILC2 can induce tissue collagen deposition (21, 39).

**Figure 2:**
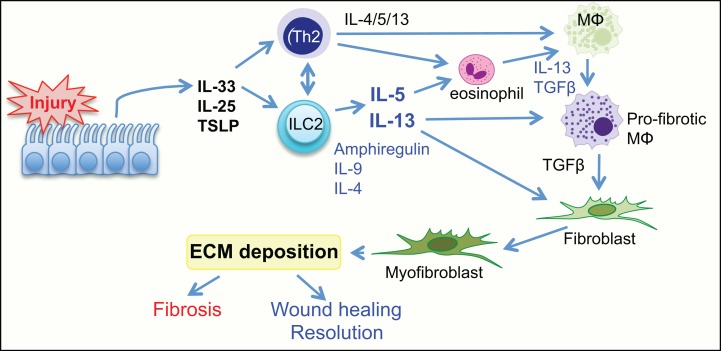
**Group 2 innate lymphoid cells have a central role in wound healing and fibrosis**. Tissue injury initiates the release of the alarmin cytokines IL-25, IL-33, and TLSP from the epithelium. This activates ILC2 and Th2 cells to release the cytokines IL-4, IL-5, IL-13, also amphiregulin, and IL-9. The release of Th2 cytokines actively promotes activation of resident macrophages to a profibrotic phenotype, induces eosinophils to release the profibrotic cytokines IL-13, PDGF, and TGFβ, and can also directly influence differentiation of fibroblasts to myofibroblasts. TSLP, thymic stromal lymphopoetin; TGFβ, transforming growth factor β; ILC2, group 2 innate lymphoid cell; MΦ, macrophage.

Increased localized expression of IL-25 and IL-33 is associated with expansion of ILC2 that may thereby promote tissue fibrosis through a number of mechanisms (Figure 2). ILC2-derived IL-5 can recruit and activate eosinophils, contributing to tissue inflammation (79). ILC2 can also enhance Th2 responses, either indirectly via IL-13-mediated DC priming or directly through major histocompatibility complex class II (MHCII) interaction with TCR on CD4^+^ T cells (56, 80, 81). ILC2-derived IL-13 can activate macrophages toward a profibrotic phenotype and can also induce collagen deposition from fibroblasts (21). These studies clearly demonstrate an important pathogenic role for ILC2 in the generation of fibrosis. This suggests that targeting ILC2 and the associated signaling pathways offers the possibility for therapeutic exploitation.

## Type 2 Responses in Fibrosis

CD4^+^ Th1 and Th2 cells and the cytokines they produce are important mediators in the inflammatory phase of fibrosis. While Th1-derived IFNγ inhibits fibrosis, the Th2 cytokines IL-4, IL-5, and IL-13 have been linked to a number of fibrotic conditions. Both IL-4 and IL-13 can promote polarization of macrophages to an alternatively activated profibrotic phenotype, recruit innate cells, such as basophils and eosinophils, and can directly act on fibroblasts to induce myofibroblast differentiation and collagen deposition (82, 83). Indeed, transgenic mice overexpressing IL-13 spontaneously develop tissue fibrosis with significant collagen deposition (84). IL-5 release by Th2 cells can also recruit and activate eosinophils, which are a potent source of the profibrotic cytokines TGF-β, PDGF, and IL-13 (85).

Studies using IL-4- and IL-13-deficient mice (*Il4*^−/−^, *Il13*^−/−^, *Il4ra*^−/−^, and *Il-13ra1*^−/−^) demonstrate a prominent role for IL-13 over IL-4 in the Th2-induced generation of fibrosis (86–89). Using IL-13-deficient mice, a profibrotic role for IL-13 was shown in *S. mansoni* egg-induced fibrosis in the livers of infected mice as well as in the lungs of egg-injected animals (87, 90). As reported first by Wynn and colleagues using soluble IL-13Ralpha2-Fc (86), a specific role for IL-13 in fibrosis was identified with anti-IL-13 antibodies now in clinical trials for fibrotic diseases (91). The functional receptors for IL-13, IL-4Rα, and IL-13Rα1 are expressed on fibroblasts, fibrocytes, and myofibroblasts (92). IL-13 can directly induce inhibition of the matrix metalloproteinase synthesis and can drive the differentiation of resident fibroblast and circulating fibrocytes to myofibroblasts, resulting in enhanced collagen deposition (83, 93, 94). These studies clearly demonstrate the importance of Th2 cells and specifically the associated cytokines, IL-4 and IL-13, in the pathogenesis of fibrosis.

**Figure 3:**
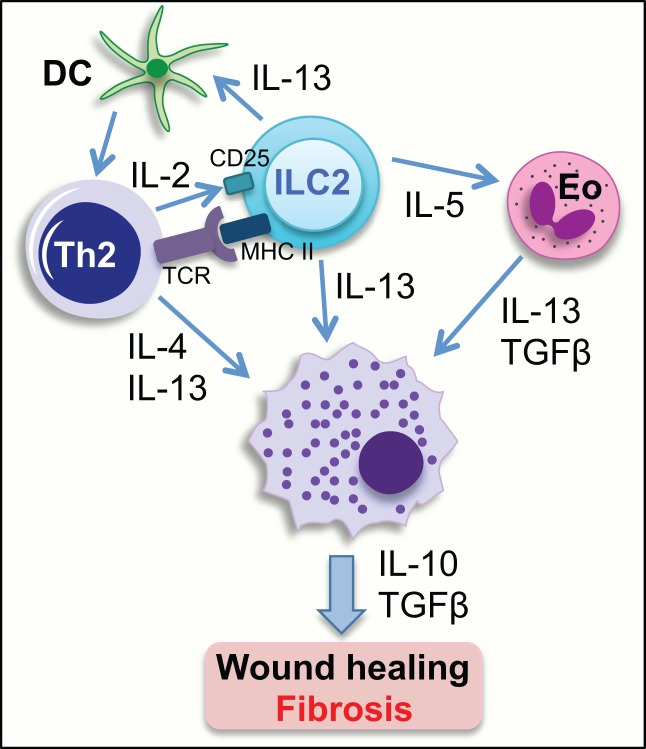
**Group 2 innate lymphoid cells can activate other cell types to initiate a wound-healing response**. ILC2 can activate Th2 cells directly through interaction between MHC class II, expressed on the surface of ILC2, and the TCR on CD4^+^ Th2 cells, and indirectly via IL-13-mediated activation of DCs. IL-2 release from Th2 cells activates IL-13-producing ILC2s via interaction with CD25 expressed on ILC2. IL-5 release from ILC2 can activate eosinophils to release IL-13 and TGFβ. IL-4 and IL-13 released from activated Th2 cells, and also ILC2- and eosinophil-derived IL-13 can activate tissue resident and infiltrating macrophages to initiate a wound-healing response, which if excessive, can result in the generation of a fibrotic lesion. Eo, eosinophil; DC, dendritic cell; TCR, T cell receptor; MHCII, major histocompatibility complex class II.

Recent studies have identified crosstalk between the innate and adaptive immune responses as integral in the initiation and maintenance of type 2 immunity (Figure 3). ILC2 is able to activate Th2 cells via MHCII-mediated antigen presentation, whereas MHCII expressing ILC3 suppresses T cell activation due to the lack of costimulatory molecules (80, 95). Antigen-specific interaction between ILC2 and Th2 cells leads to the production of IL-4, IL-13, and also IL-2 by the Th2. Notably, Th2-derived IL-2 interacts with CD25 expressed on ILC2 activating ILC2 to release IL-13 (80). Furthermore, in addition to directly producing IL-13, ILC2 produces IL-5, which activates eosinophils, which are also potent producers of IL-13 and TGF-β (79). These cytokines are all able to activate recruited and resident macrophages to a profibrotic phenotype, as well as directly inducing trans-­differentiation of fibroblasts. This interplay between innate ILC2 cells and adaptive CD4^+^ T cells to induce macrophage activation and myofibroblast differentiation provides interesting mechanistic insight and identifies pathways that could potentially be exploited by novel therapeutics.

## Macrophages Subtypes, Inflammation, and Fibrosis

**Figure 4:**
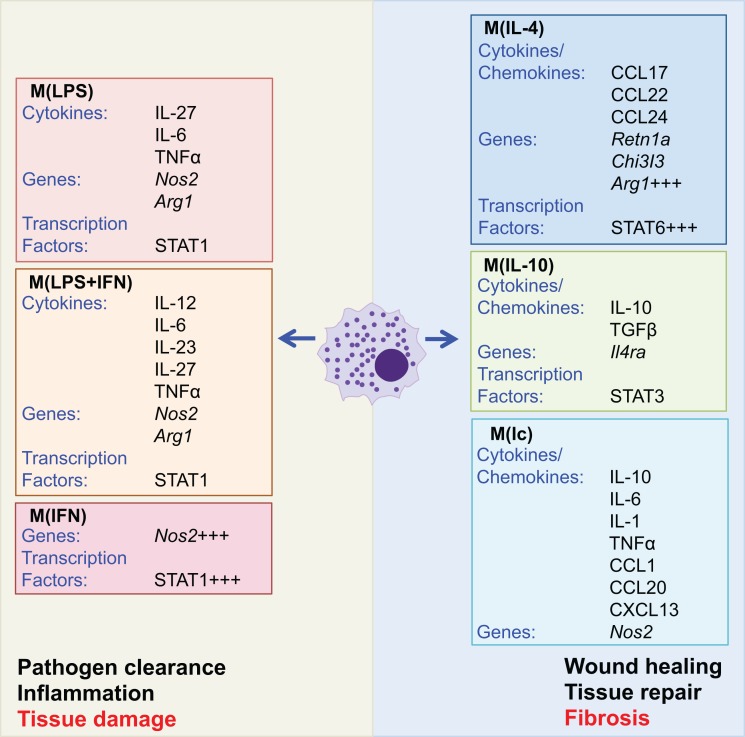
**Activated macrophage subtypes**. Macrophage subtypes can be described by the experimental stimulus used to activate the cells. Although there are multiple macrophage subtypes, these can broadly be split into two groups associated with inflammation and pathogen clearance or wound healing and tissue repair. Exacerbated activation of both groups has the potential to cause pathological tissue damage or fibrosis (outlined in red text). Macrophages activated with lipopolysaccharide (LPS), interferon (IFN) γ, or a combination of the two (LPS + IFN) are associated with increased expression of Nos2 and production of proinflammatory cytokines. Conversely, activation with IL-4 and IL-10 increases expression of IL-10, TGFβ, and arginase (Arg1), while activation with immune complexes (Ic) gives a phenotype similar to that elicited by LPS. However, activation by IL-4, IL-10, and immune complexes is associated with tissue repair and wound healing (98).

Macrophages are phagocytic cells, which are integral in homeostasis, development, and immunity and are found in all tissues where they display distinct anatomical and functional diversity. A brief overview of the central role that macrophages play in fibrosis is provided, as there have recently been a series of comprehensive reviews focused on macrophages (1, 96–99). Resident macrophages regulate tissue homeostasis by responding to changes in the local environment. If required, circulating monocytes are recruited to the site of insult and activated to the desired phenotype or resident cells may proliferate locally in response to tissue injury (100). Macrophages can exist in many activation states dependent upon the inflammatory environment or stimulation used (98). Macrophages were commonly broadly divided into two subtypes: those associated with a type 1 response, termed “classically” activated or “M1,” which are generally proinflammatory, and “alternatively” activated or “M2,” which are typically associated with type 2 responses and wound healing. These two macrophage subtypes are defined experimentally by *in vitro* responses to IFNγ and the TLR4 agonist lipopolysaccharide (LPS) and the Th2 cytokines IL-4 and IL-13, respectively, with macrophages differentially generated having a unique gene profile and distinct functions. However, it is now accepted that the broad M1 versus M2 dichotomy terminology does not adequately describe the diverse phenotypes of macrophages. Therefore, newer and broader characterization of subtypes based on the activation of the macrophages under experimental conditions has been proposed (Figure 4) (98). Macrophages have a key role in the generation of fibrosis with distinct subtypes temporally activated and expanded in damaged tissue contributing to aspects of both the development of fibrosis and its subsequent resolution (97). Studies specifically depleting CD11b^+^F4/80^+^ macrophages, using *Cd11b*-DTR mice, have demonstrated that macrophages are crucial for the maintenance of type 2 immunity and also the associated generation of fibrosis (101, 102).

When tissues are damaged following infection or injury, circulating Ly6C^+^ monocytes are recruited and differentiate into proinflammatory macrophages as they migrate through the affected tissue (103). Proinflammatory macrophages elicited via STAT1 in response to localized release of IFNγ or TLR agonists are a potent source of the cytokines tissue necrosis factor (TNF)-α, IL-6, IL-12, and IL-23, and reactive oxygen species (ROS), which act to kill invading pathogens (96). To counteract the damaging effects of macrophage-derived reactive oxygen and nitrogen species to the local tissue, macrophages undergo apoptosis or switch to an anti-inflammatory phenotype, which dampens the immune response and facilitates tissue repair (96). If the causal insult is not removed, as is the case in a number of chronic inflammatory diseases, the resulting aberrant activation of macrophages can lead to fibrosis. Indeed, macrophages play a crucial role in the pathogenesis of most chronic fibrotic diseases.

Activation of macrophages by proinflammatory stimuli causes a metabolic switch from oxidative phosphorylation to glycolysis, similar to the Warburg effect originally identified in tumors (104, 105). This switch occurs in response to inflammatory stimuli, such as LPS and type I interferon, as well as hypoxic conditions and activation of hypoxia-inducible factor-1α (HIF-1α) (105). Indeed, the metabolic status of macrophages is closely linked to their function. Aerobic glycolysis is initiated upon activation of proinflammatory macrophages, increasing the uptake of glucose and attenuating the activities of the respiratory chain allowing for the generation of ROS, this provides the cell with a rapid release of energy essential for the removal of pathogens (106). Conversely, anti-inflammatory macrophages have a more sustained role requiring a slower release of energy and thus rely on fatty acid oxidation and oxidative metabolism (107). There is a clear distinction in metabolism between macrophage subtypes; however, the relevance of these observations and the implications for diseases, such as fibrotic disease, are, as yet, not fully understood.

The development of anti-inflammatory macrophages within a type 2 immune environment in response to IL-4 and IL-13 via STAT6 signaling has specific functions in wound repair and resolution (99). Macrophages elicited by IL-4 and IL-13 have a distinctive expression profile characterized by high expression of Arginase (Arg) 1, chitinase-like protein Ym1 and RELMα, and release of the chemokines CCL17, CCL22, and CCL24 (Figure 4). Macrophages can also be activated by IL-10, via STAT3, which results in autocrine production of IL-10; these macrophages are characterized by expression of IL-4Rα [Figure 4 (108)]. Indeed, IL-4/IL-13-primed macrophages expressing Arg1 have been shown to inhibit IL-13-mediated fibrosis, via suppressing the activation of CD4^+^ T cells and suppressing myofibroblasts by competing for arginase in the local environment (109, 110). Conversely, IL-13-elicited macrophages are also implicated in the pathogenesis of fibrosis (102). There is clearly a balance between the pro- and antifibrotic roles of macrophages in inflammation; however, IL-13-elicited profibrotic macrophages (PFMs) are associated with the release of TGF-β and are considered profibrotic in most chronic inflammatory diseases.

Distinct from the pro- and anti-inflammatory macrophage populations a CD11b^low^ non-phagocytic macrophage population that does not express Arg1, termed resolution-promoting macrophages (Mres), has been identified in the lymphoid organs and adipose tissue (111). These macrophages appear to be antifibrotic and immune regulatory, secreting low levels of inflammatory cytokines and IL-10 and therefore may play an important role in the localized and systemic termination of an inflammatory response (112).

Recently, the epithelial-derived cytokines IL-25, IL-33, and TSLP discussed above have been shown to activate macrophages, both directly and indirectly, by promoting expansion of IL-13-expressing ILC2 (113–115). Indeed, IL-13 production from ILC2 and also eosinophils and Th2 cells has been shown to induce and maintain localized tissue macrophage activation both in the lung and in the adipose tissue (21, 115, 116). This interplay between ILC2 and Th2 cells in the maintenance of potentially PFMs at tissue sites could have implications in fibrotic disease.

Given the heterogeneity of macrophages (Figure 4), studies have focused on characterizing the PFM populations. These include IL-4-elicited proangiogenic PFMs that express a number of factors that are key mediators in the tissue repair process including TGF-β, PDGF, VEGF, as well as a number of matrix metalloproteinases (MMPs) (96). These factors contribute to the fibrotic cascade via recruitment of tissue fibroblasts, circulating fibrocytes and bone marrow-derived myofibroblasts, activation of resident myofibroblasts, and differentiation of epithelial cells into myofibroblasts through EMT. Indeed, in fibrotic tissue, macrophages localize in close proximity to myofibroblasts, suggesting the importance of macrophages and macrophage-derived mediators in the progression of fibrosis (2). Macrophages are clearly important regulators of wound healing and therefore also fibrosis. The heterogeneity in macrophage populations (Figure 4) highlights the extent of further mechanistic investigation needed to address the relative roles of macrophage populations in the fine balance between wound healing and fibrosis.

## Conclusion

In this article, we have expanded on the potential roles of innate cells in fibrosis with a focus on the interplay between the epithelial-derived cytokines, ILC2, and macrophages. We have also explored the role of ILC2 in bridging the innate and adaptive immune system in the context of inflammation and fibrosis. Dysregulation of macrophages underlies a majority of inflammatory and fibrotic disease conditions, with a number of therapies targeting macrophages currently under development (97). While the relative roles of macrophages in the induction and resolution of fibrosis have been extensively studied, it is yet unclear whether distinct populations of macrophages control these disparate functions, or whether the phenotype of the local macrophages alters dependent on changes in the tissue microenvironment. Many mechanisms underlying fibrosis are common to multiple organs, which is important for the development of potential therapeutics (117). A key to developing effective therapeutics for tissue fibrosis is the identification of common pathways and, although further studies are needed, the epithelial cytokines and ILC2 axis interplay with macrophages is a promising area for therapeutic intervention.

## Conflict of Interest Statement

The authors declare that the research was conducted in the absence of any commercial or financial relationships that could be construed as a potential conflict of interest.
